# Exploring the Application of Terahertz Metamaterials Based on Metallic Strip Structures in Detection of Reverse Micelles

**DOI:** 10.3390/bios14070338

**Published:** 2024-07-11

**Authors:** Ziqin Fu, Jin Chen, Xiangxue Chen, Yu Sun, Fengchao Wang, Jing Yang

**Affiliations:** College of Science, Shanghai Institute of Technology, 100 Haiquan Road, Shanghai 201418, China; 216182104@mail.sit.edu.cn (Z.F.); jinchenxl@sit.edu.cn (J.C.); 226182100@mail.sit.edu.cn (X.C.); yusunw@sit.edu.cn (Y.S.); fcwang@sit.edu.cn (F.W.)

**Keywords:** terahertz spectroscopy, metamaterial sensor, reverse micelles

## Abstract

Terahertz spectroscopy has unique advantages in the study of biological molecules in aqueous solutions. However, water has a strong absorption capability in the terahertz region. Reducing the amount of liquid could decrease interference with the terahertz wave, which may, however, affect the measurement accuracy. Therefore, it is particularly important to balance the amount and water content of liquid samples. In this work, a terahertz metamaterial sensor based on metallic strips is designed, fabricated, and used to detect reverse micelles. An aqueous confinement environment in reverse micelles can improve the signal-to-noise ratio of the terahertz response. Due to “water pool” trapped in reverse micelles, the DOPC (1,2-dioleoyl-sn-glycero-3-phosphocholine) solution and DOPC emulsion can successfully be identified in intensity by terahertz spectroscopy. Combined with the metamaterial sensor, an obvious frequency shift of 30 GHz can be achieved to distinguish the DOPC emulsion (5%) from the DOPC solution. This approach may provide a potential way for improving the sensitivity of detecting trace elements in a buffer solution, thus offering a valuable toolkit toward bioanalytical applications.

## 1. Introduction

Terahertz (THz) spectroscopy has potential applications in investigating the characteristics of biological samples, such as proteins, carbohydrates, DNA, and cells, due to its specific vibrational modes and unique characteristics of low photon energy level [1,2,3]. Water serves as the medium and solvent for the vast majority of physical and biological processes, which is crucial for sustaining various life-sustaining biological processes [4], but the strong absorption of terahertz waves in water makes it hard to extract information in aqueous solutions [5]. This significantly limits applications of terahertz technology in the biological field.

Metamaterials are periodic artificially designed electromagnetic structures and may enhance the interaction between the terahertz wave and the target molecules to improve sensitivity [6]. In addition, THz functional devices with different features can be designed based on metamaterials with different electromagnetic structures [7,8,9]. The THz metamaterial sensing technique is appropriate for applications concerning biochemical sample detection [10,11], such as drugs [12], proteins [13], DNA [14,15,16], and RNA [17,18,19], by harnessing the localized surface plasmon resonance effect and the specific absorption characteristics of different substances. For example, Tao et al. demonstrated a SRR metamaterial with an ultrathin substrate for detecting protein thin films of various thickness [20]. Shih et al. demonstrated a nanofluidic THz metamaterial sensing platform to detect different alcohols in water solutions [21]. Du et al. proposed a solution analysis platform based on a double-formant metamaterial resonator to detect plant growth regulators [22]. Cui et al. designed terahertz metamaterial biosensors based on double spilt-ring resonators for quantitatively evaluating the level of detection of carcinoembryonic antigens [23]. The combination of terahertz waves and metamaterials has provided new insights for the development and application of sensing technologies [24,25,26]. It not only features non-destructive and rapid response characteristics but also enhances detection limits and resolution. Nevertheless, there are still unresolved problems in THz metamaterial sensing systems for detecting biological samples, such as the control of homogeneous samples and dependency on a small volume of an aqueous solution. Moreover, integrating metamaterials with microfluidic channels [21,27], graphene [28,29], and photonic crystals [30] has been demonstrated for an efficient sensor for label-free sensing applications. According to this inspiration of metamaterials coupled with others, we tried to integrate water nanodroplets with metamaterials.

A water-in-oil emulsion consists of water nanodroplets dispersed in a lipid continuous phase, stabilized by surfactant molecules [31]. Reverse micelles can be easily self-assembled by amphiphilic hydroperoxides and some other surface-active compounds in a nonpolar solvent, which look like nanometer-scale cages and trap the water and hydrophilic components in a nanoscale “water pool” [32,33,34]. The self-assembledstructure provides a cell-like model for a physiological environment, which can fulfill a number of the requirements for a life-mimicking system [35]. Reverse micelles are widely used as a mode system for studying aqueous solutions in confined environments [36,37,38]. For example, Murakami et al. carried out terahertz absorption spectroscopy for AOT reverse micellar solutions with and without myoglobin [39]. Yang et al. proposed a novel strategy to probe the terahertz dynamics of the interaction of metal ions and lipids in phospholipid reverse micelles [40]. Tang et al. developed a label-free optical platform for detecting the hydration dynamics of collective Aβ aggregates in water-in-oil nanodroplets by using terahertz spectroscopy [41]. Zhang et al. measured the terahertz refractive index of living cells in near-physiological environments by usingcell-containing droplets [42]. The water-in-oil structure not only provides a cell-like near-physiological environment, but improves the signal-to-noise ratio of the terahertz response due to the confinement environment of aqueous solutions.

In this work, reverse micelles are introduced into terahertz metamaterial sensors to explore aqueous solutions. We experimentally demonstrated a terahertz band-stop filter based on a metallic strip metamaterial structure. The resonance frequency and modulation depth of the filter can be tuned by varying the length and direction of the metallic strips, respectively. In addition, we investigated its potential applications in refraction index sensing. When the metamaterial sensor was applied, liquids with similar absorption properties and different dielectric properties could be distinguished by the resonance peak shift. Furthermore, we used reverse micelles to provide an aqueous environment, and the “water pool” trapped in reverse micelles was sensitive to terahertz waves, which may recognize the slight differences between the DOPC solution and DOPC emulsion. The combination of reverse micelles and metamaterials offers a valuable toolkit toward bioanalytical applications in terahertz biology.

## 2. Materials and Methods

### 2.1. Reverse Micelles

In the experiments, DOPC (1,2-dioleoyl-sn-glycero-3-phosphocholine) as a surfactant and anhydrous hexadecane as nonpolar solvent were sonicated to prepared the reverse micelle emulsion. Millipore water, as a kind of dispersed phase, was used to prepare all the buffers, solutions, and emulsions. The phospholipids were dissolved in chloroform and evaporated with nitrogen gas in order to obtain the thin film. Then, they were dissolved in hexadecane to obtain definite concentration. After that, millipore water was injected in the mixed solution of phospholipid and hexadecane. Furthermore, the homogeneous reverse emulsions could be prepared after gently warming (25 °C) and a series of vortexes and sonicates. The sample looks like a white, milky liquid (Figure S1).

### 2.2. THz Spectroscopy

A transmitted terahertz time-domain spectroscopy (THz-TDS) system (Figure S2) was applied to measure the samples to obtain the optical constants. A Mai Tai femtosecond fiber laser delivers pulses with central wavelength of 800 nm, pulse width of 100 fs, and repetition frequency of 80 MHz, which are divided into a pump beam for THz generation and a probe beam for THz detection. The optical path difference between the pump beam and probe beam can be precisely controlled by the electric translation platform, so the probe beam can be manipulated to obtain the complete THz time domain spectra through software programming. Like our previous work [9], the analytes were placed in fused quartz cuvettes, which were fixed on the sample holder in the THz-TDS. The setup was enclosed in a chamber which was purged with dry air-purged (RH ≈ 1.2%) and temperature-controlled conditions (21.5 ± 0.5 °C).

Through fast fourier transformation (FFT) of the measured THz time domain spectra, both the phase and power as a function of the frequencies can be resolved. Subsequently, the transmission (dB) as a function of frequency can be derived as follows:

$$T\;(dB)=10\mathrm{lg}(P_{sam}/P_{ref})$$

where *P_sam_* and *P_ref_* are the power of sample and reference, respectively.

### 2.3. Design of Metamaterial Structure

**Figure 1:**
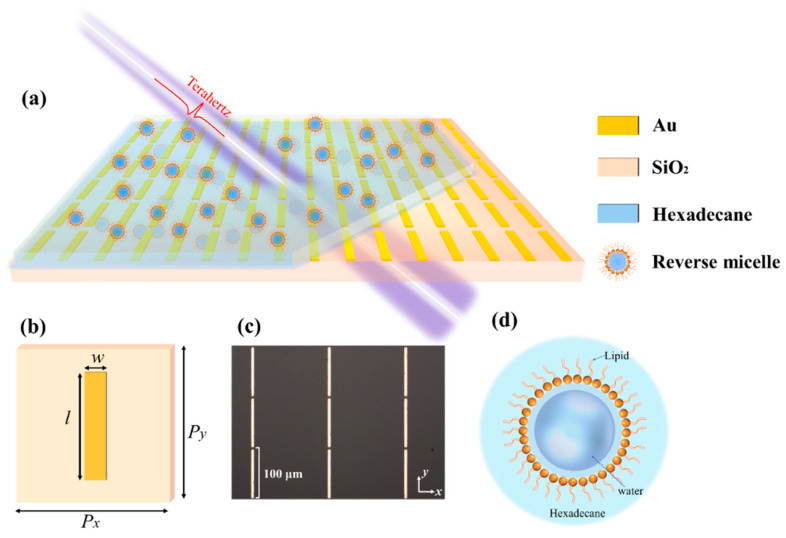
(**a**) Schematic of the terahertz metamaterial sensor impinged by the terahertz probe. (**b**) Image of a unit cell. (**c**) Partial photomicrograph of the fabricated metamaterial sample with the length *l* = 95 μm. (**d**) Illustration of reverse micelles formed by phospholipids.

Figure 1a depicts a schematic diagram of the proposed metamaterial composed of metallic strips for biological detection. The metamaterial consists of two layers: the substrate is made of fused quartz with the relative permittivity *ε*_r_ = 3.92, and the metallic (Au) strips with the conductivity *σ* = 4.56 × 10^7^ S/m is deposited on the substrate. Thickness of the quartz and metal is 500 μm and 200 nm, respectively. As shown in Figure 1b, the structural parameters are as follows: *P*_x_ = 150 μm, *P*_y_ = 100 μm, *l* = 95 μm, and *w* = 5 μm. The designed metamaterial was fabricated using electron beam etching. The partial microscopy image of the fabricated metamaterial sample can be seen in Figure 1c. In the tests combining metamaterial, the fused quartz cuvette will be improved by attaching a metamaterial sample to the inner wall of the cuvette. Our photonic biosensor used phospholipid reverse micelles to provide an aqueous environment for biological detection, as depicted in Figure 1d.

To validate the terahertz spectral response of the designed metamaterial, numerical simulations based on CST Microwave Studio 2021 were performed. In the numerical modeling, the magnetic boundary conditions were applied along the *x*-direction, the electric boundary conditions were applied along the *y*-direction, while the open boundary conditions were used along the *z*-axis. Various field monitors were employed to monitor the distribution of electric fields, magnetic fields, and surface currents at frequency locations of the resonance peaks. The data output from the field monitors provided intuitive insights into the distribution characteristics of the electromagnetic fields within the simulated structure, aiding in analysis and design optimization. Based on transmission spectra, the metamaterial performances of the unit cell were calculated and analyzed.

## 3. Results

### 3.1. Effect of the Length of Metallic Strips

**Figure 2:**
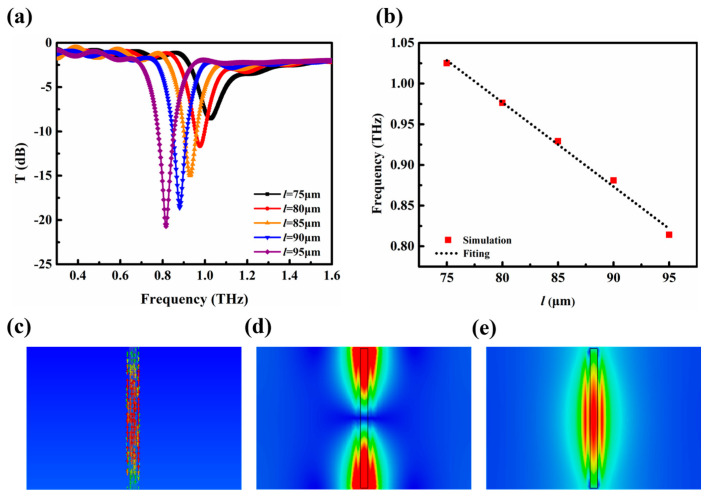
(**a**) Terahertz transmission spectra of metal strips with different lengths for the *y*-polarized incident terahertz beam. (**b**) Variation in transmission with different lengths. (**c**) Current distribution. (**d**) Electric and (**e**) magnetic fields on the metallic strip at the resonance frequency 0.81 THz.

To simulate the influence of the length of metallic strips on the resonance characteristics of the metamaterial, metamaterials with different metallic strips lengths were designed and simulated. The numerically simulated transmission spectra of the metamaterials with different metallic strip lengths are indicated in Figure 2a. When the length decreases from 95 μm to 75 μm, the resonance peak shifts from 0.81 THz to 1.03 THz, and the resonance intensity can also be improved from −9 dB to −21 dB. Figure 2b (red squares) represents the numerical result of the resonance frequency for *l* = 75, 80, 85, 90, and 95 μm. As can be seen, by increasing the metallic strip length, one finds a linear decrease in the resonant frequency. The linear relationship between the resonant frequency and length of the metallic strip can be explained by a Fabry–Perot-like model [43,44]. Here, due to the width of the metallic strips being much less than their length, the metallic strips can be treated as the equivalent of cavities for the surface charge wave, and the resonant frequency is in inverse proportion to the metallic strip length [43,45]. The resonance frequency can be written as $f=c/2n_{eff}l$, where *f* is the resonance frequency, *c* is the speed of propagation of the terahertz wave, *n_eff_* is the background effective index, and *l* is the metallic strip length. From this relationship, one finds an inverse relation between the metallic strip length and resonance frequency.

In Figure 2c–e, the current distribution and the electric and magnetic field intensity at the resonance frequency 0.81 THz (*l* = 95 μm) were simulated. As shown in Figure 2c, the surface current is mainly distributed on the metallic strip, exciting an electric dipole mode. In Figure 2d,e, the electric field is mainly concentrated at both ends of the metallic strip, while the magnetic field is mainly distributed around the middle of the metallic strip. This is because metallic strip structures can succeed in combining strong localization and high field enhancement in the THz region [43,44,46]. Therefore, the metallic strip structure is promising due to the strong enhancement of the electromagnetic field, tunability of resonant frequency, and simplicity of structure.

### 3.2. Effect of the Rotation of Metallic Strips

**Figure 3:**
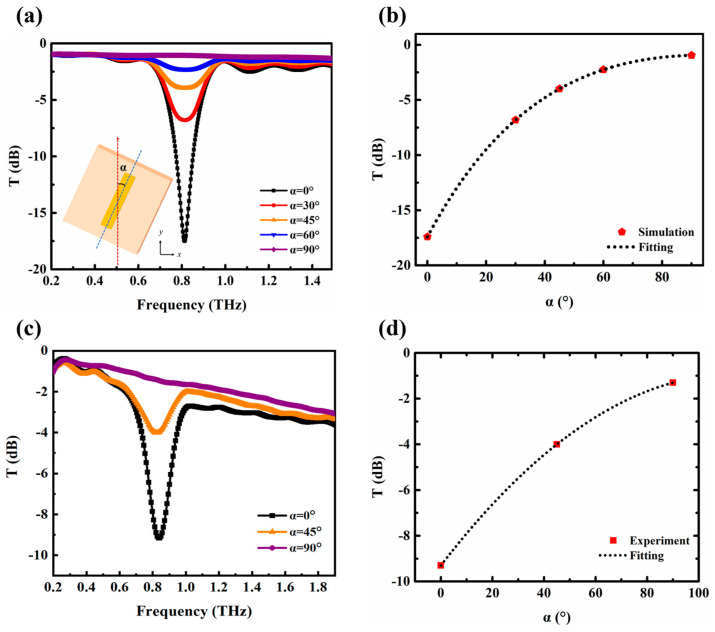
(**a**) Simulated and (**c**) experimental terahertz transmission spectra at different *α* angles. (**b**) Simulated and (**d**) experimental variations in transmission with different *α* angles.

In the simulations, we selected a metallic strip length of 95 μm for further investigation of the resonance characteristics. The propagation and response of the terahertz waves were controlled by the structure of the metamaterials, where one crucial parameter is the polarization angle. As shown in the insert of Figure 3a, the angle *α* between the long axis of the metallic strips and the polarization direction of the incident THz waves was taken into account. Under the normal incident THz beams with a polarization direction along the *y*-axis, the angle α was adjusted by rotating the metallic strip structure to further investigate the resonant characteristics. As illustrated in Figure 3a, a remarkably sharp dipole resonance valley occurs at 0.81 THz in the transmission spectrum when α is 0 degrees, achieving a filtering efficiency of over 99.9%. This can be attributed to the excitation of surface plasmon polaritons when the incident electromagnetic wave interacts with the metal surface, resulting in resonant phenomena at specific frequencies. Figure 3b summarizes the relationship between α and the resonance intensity. As α increases, the filtering intensity of the resonance peak significantly decreases, while the corresponding frequency remains almost constant. Since the rotation of the metal strip reduces its effective length in the *y*-axis direction, the coupling between the electric field of the terahertz wave and the structure of the metallic strip metamaterial is weakened, resulting in the attenuation of the polarization effect.

In order to experimentally verify the angle tunability characteristic of the designed filter, the single metallic strip structure with a 95 μm length was fabricated using electron beam etching, and a microscope photograph of the sample is depicted in Figure 1c. During the experiment, firstly, the metallic strip metamaterial was positioned in the THz-TDS system and the long axis of the metallic strips was made parallel to the *y*-axis (the polarization direction of the incident THz waves), in which case, the angle *α* is 0 degrees. There exists a sharp resonance peak at 0.83 THz with a quality factor of 16.1. Then, we rotated the metamaterial sample to alter the angle *α* between the long axis of the metallic strips and the *y*-axis, and the corresponding terahertz transmission spectra were recorded in Figure 3c. As *α* varied from 0 to 45 degrees, the transmission intensity at the resonance peak increased from −9.1 dB to −4.0 dB, and when the angle *α* increased to 90 degrees, the transmission intensity was almost zero. It can be observed that the experimental results are in good agreement with the simulation results, indicating that the prepared metallic strip metamaterial can be used as tunable terahertz filters.

### 3.3. THz Spectroscopy Detection of Refractive Index Liquids Using Metamaterial Sensor

**Figure 4:**
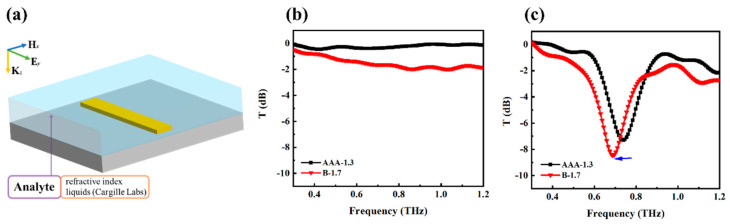
(**a**) Schematic diagram of refractive index liquids in an improved cuvette (with metamaterial). Terahertz transmission spectra of AAA-1.3and B-1.7 (**b**) without and (**c**) with metamaterial. The blue arrow represents a shift in resonance frequency of AAA-1.3 and B-1.7.

To demonstrate the sensing potential of the designed THz metamaterial filter, we tested whether the combination of metamaterial could improve the sensitivity. Two types of refractive index liquids (Cargille Labs, Cedar Grove, NJ, USA) were used in the experiments:AAA-1.3 (*n* = 1.3) and B-1.7 (*n* = 1.7). In the experiments, the liquid samples were placed in fused quartz cuvettes with 1 mm thickness, and the empty cuvettes worked as a reference signal. Figure 4a shows the schematic diagram of refractive index liquids in an improved cuvette (with metamaterial). For comparison, the measured transmission spectra of AAA-1.3and B-1.7without and with metamaterial are shown in Figure 4b,c. It can be observed from Figure 4b that the terahertz transmission spectra for both the refractive index liquids exhibit a slight difference in intensity, due to the minor composition differences between the AAA-type and B-type refractive index liquids. But the experimental results lack distinctive spectral characteristics, making it a challenge to distinguish the two substances. Importantly, when the refractive index liquids were placed in a liquid cell with the metamaterial, the THz transmission spectra could be distinguished by the response peak shifting from to 0.74 THz (AAA-1.3) to 0.68 THz (B-1.7), as depicted in Figure 4c. In contrast to the ambiguous signals of the two refractive index liquids in an empty cell, the transmission spectra in a liquid cell with metamaterial can unambiguously distinguish the two types of liquids. The change in the refractive index of the analyte around the resonance of the metamaterial is converted into the change in the field distribution at the resonance point, causing the resonant frequency shift. Therefore, when the metamaterial sensor was applied, liquids with similar absorption properties could be distinguished by the resonance peak shift rather than by absorption.

### 3.4. THz Spectroscopy Detection of Integrating Metamaterial Sensor and Reverse Micelles

**Figure 5:**
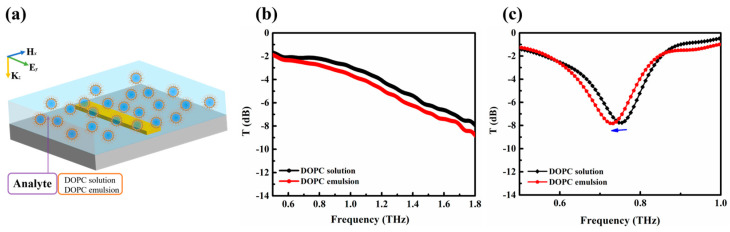
(**a**) Schematic diagram of the metamaterial structure integrated with reverse micelles in a cuvette. Terahertz transmission spectra of the DOPC solution and DOPC emulsion (**b**) without and (**c**) with metamaterial. The blue arrow represents a shift in resonance frequency of DOPC solution and DOPC emulsion.

A terahertz sensor integrating metamaterials and reverse micelles is demonstrated in the following. In the experiments, the DOPC solution refers to the mixture of the phospholipid/hexadecane solution, while the DOPC emulsion is a kind of reverse micelles prepared by phospholipid (DOPC), hexadecane, and millipore water (5%). In this part, the liquid samples were placed in fused quartz cuvettes with 0.7 mm thickness, and the empty cuvette works as a reference signal. Figure 5a shows the schematic diagram of the metamaterial structure integrated with reverse micelles in a cuvette. Figure 5b,c show the terahertz transmission spectra of the DOPC solution and DOPC emulsion without and with metamaterial, respectively. Figure 5b shows the obtained terahertz transmission spectra of the DOPC solution and DOPC emulsion. It can be seen that two spectra exhibit a slight difference in intensity, which may be caused by a small amount of water in the DOPC emulsion. Therefore, the “water pool” trapped in reverse micelles can be used as a probe for studying water and hydrophilic components in terahertz biology. Further, when the liquid samples were injected into an improved cuvette (with metamaterial), the terahertz transmission spectra could be distinguished by the response peak shifting from to 0.75 THz (DOPC solution) to 0.72 THz (DOPC emulsion). These results indicate that compared to using only the “water pool” in reverse micelles, the integration of metamaterials and reverse micelles could further improve the sensitivity of external target detection by the resonance peak shift. In our previous work [40], the terahertz dynamics of the “water pool” confined in phospholipid reverse micelles were developed as a probe to explore the interactions of metal ions with lipid membrane interfaces. In this work, we chose a simple platform by integrating reverse micelles and metamaterials to enhance the signal-to-noise ratio of the terahertz response. The investigation of reverse micelles under terahertz metamaterials will inspire future work for the ultrasensitive detection of metal ions in liquids.

## 4. Conclusions

We have demonstrated a label-free terahertz sensor integrating metamaterials with reverse micelles. A single metallic strip structure was proposed for sensing applications, and the resonant characteristics of the metallic strips with different lengths and orientations were investigated. The resonance frequency shifts from 0.81 THz to 1.03 THz when the length of the metallic strips decreases from 95 μm to 75 μm, and the transmission of the resonance peak increases from −9.1 dB to −0.8 dB when the angle α between the long axis of the metallic strips and the polarization direction of the incident THz waves increases from 0 to 90 degrees. In addition, we investigated its potential applications in refraction index sensing. When the metamaterial sensor was applied, two types of refractive index liquids (AAA-1.3 and B-1.7) with similar absorption properties could be distinguished by the resonance peak shifting from 0.74 THz to 0.68 THz. Furthermore, we used reverse micelles to provide the confinement space to explore an aqueous solution. As there are the slight differences between the DOPC solution and DOPC emulsion in the “water pool”, the resonance peaks of which are quite distinct from each other in the resonance frequency. This approach paves the way to build ultrasensitive THz sensors for aqueous solutions that can offer potential applications in the bioanalytical field.

According to previous works, the combination of terahertz waves and metamaterials provides a new detection method for biomedical molecules, which can not only achieve label-free detection, but also refresh the resolution limit of existing sensors. However, one of the major hurdles for biological applications lies in the poor THz transmission of polar liquids, such as water, which makes it difficult to extract information about solute molecules. In this work, we focus on how to balance the amount and water content of liquid samples in terahertz biosensing applications. Metamaterials coupled with others may be very promising routes for the highly sensitive THz spectroscopy detection of biological samples in the future. Reverse micelles can be formed by the self-assembly of surfactant molecules in a nonpolar solvent and become a nanometer-scale spherical cage filled with water, which are considered a good model of cells, and have been proven to be a suitable tool to study dielectric properties in the THz region. Reverse micelles are introduced into terahertz metamaterial sensors to explore aqueous solutions, which can overcome the strong absorption issues under physiological aqueous conditions and realize biosensing of water-based substances. Therefore, we chose a new platform by integrating reverse micelles and metamaterials to enhance the signal-to-noise ratio of the terahertz response. We do believe that the investigation of “Metamaterials+” will inspire our future work in terahertz sensing.

## Data Availability

The data generated an analyzed in this study are available from the corresponding author on reasonable request.

## Supplementary Materials

The following supporting information can be downloaded at: https://www.mdpi.com/article/10.3390/bios14070338/s1, Figure S1: A sample photo of DOPC reverse emulsions; Figure S2: (a) A schematic diagram and (b) a photo of THz-TDS experimental setup.
