# Supplementary material for: Exploring the Application of Terahertz Metamaterials Based on Metallic Strip Structures in Detection of Reverse Micelles
Source: Biosensors (Basel). 2024 Jul 11;14(7):338. doi: 10.3390/bios14070338 (PMC11275120; doi:10.3390/bios14070338)
Supplement: Supplementary file 1 [file biosensors-14-00338-s001.zip › biosensors-3011591-supplementary.pdf]

biosensors

Supplementary Material

Exploring the Application of Terahertz Metamaterials Based on Metallic Strip Structures in Detection of Reverse Micelles

Ziqin Fu †, Jin Chen †, Xiangxue Chen, Yu Sun, Fengchao Wang and Jing Yang *

College of Science, Shanghai Institute of Technology, 100 Haiquan Road, Shanghai 201418, China; 216182104@mail.sit.edu.cn (Z.F.); jinchenxl@sit.edu.cn (J.C.); 226182100@mail.sit.edu.cn (X.C.); yusunw@sit.edu.cn (Y.S.); fcwang@sit.edu.cn (F.W.)

* Correspondence: yangjingxqq@sit.edu.cn

† These authors contributed equally to this work.

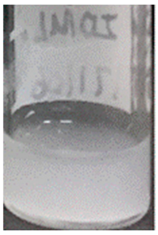

Figure S1. A sample photo of DOPC reverse emulsions.

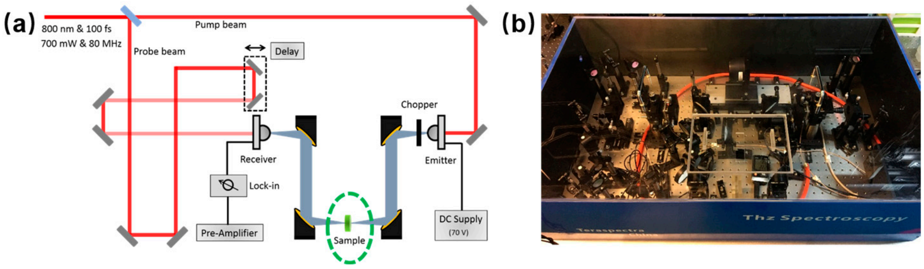

Figure S2. (a) A schematic diagram and (b) a photo of THz-TDS experimental setup.
